# Enhancing formation rate of highly-oriented silicon nanowire arrays with the assistance of back substrates

**DOI:** 10.1038/s41598-017-03498-y

**Published:** 2017-06-09

**Authors:** Chia-Yun Chen, Ta-Cheng Wei, Cheng-Ting Lin, Jheng-Yi Li

**Affiliations:** Department of Materials Science and Engineering, National Cheng Kung University, Tainan, 701 Taiwan

## Abstract

Facile, effective and reliable etching technique for the formation of uniform silicon (Si) nanowire arrays were realized through the incorporation of back substrates with metal-assisted chemical etching (MaCE). In comparison with conventional MaCE process, a dramatic increase of etching rates upon MaCE process could be found by employing the conductive back substrates on p-type Si, while additionally prevented the creation of nanopores from catalytic etching reaction. Examinations on the involving etching kinetics, morphologies, wetting behaviors and surface structures were performed that validated the role of back substrates upon MaCE process. It was found that the involved two pathways for the extraction of electrons within Si favored the localized oxidation of Si at Si/Ag interfaces, thereby increasing the etching rate of MaCE process. This back-substrate involved MaCE could potentially meet the practical needs for the high-yield formation of Si nanowire arrays.

## Introduction

Silicon (Si)-based solar cells, with more than 87% of market share, dominated the current photovoltaic technology in industry. Recently, it has been intensively explored that the introduction of low-dimensional Si nanostructures, such as nanowires or nanorods on cell architectures, could greatly improve the light-absorption of Si-based solar cells covering the broadband visible wavelengths, which was potential for the advanced development of high-performance solar cells^1, 2^. In addition, Si nanowires, in particular aligned or ordered organizations, displayed exceptional electric, photonic and chemical properties and have been explored as potential building blocks for numerous functional devices, including circuit elements^3^, Microelectromechanical systems (MEMs) devices^4^ and biological sensors^5, 6^. Various methods have been made to prepare aligned Si nanowire arrays, such as vapor-liquid-solid growth^7^, oxide-assisted growth^8^, laser ablation^9^, epitaxial growth^10^ and others. However, high growth temperatures were inevitably involved in the synthetic processes. Also, it remained difficulties in controlling both of diameters and doping levels of formed nanowires, which hindered their practical application for commercialized products.

Recently, metal-assisted chemical etching (MaCE) has been proposed for nanowire fabrication with advantages including low-cost, room-temperature and large-area capabilities, which benefited many nanowire-based functional devices^11–16^. Involvement of MaCE process required noble metals as catalyst for electrochemical reaction, such as gold and silver. The catalytic etching with predefined metallic catalysts was initiated with the hole injections from oxidative agents through the metal/Si interfaces that oxidized Si atoms right beneath the metallic catalysts. Subsequently, the oxidized Si was dissolved away with hydrogen fluoride (HF) etchants. Penetration of metallic catalysts into Si substrates caused the etching pores vertically to the substrate planes, leaving the remnant Si as nanowire structures. The involving kinetic processes, along with the formation rate of Si nanowires, were dominantly determined by the reaction temperature and concentration of oxidants^17, 18^. Dependence of etching rate on the reaction temperature has been systematically studied, which indicated the etching rates of MaCE was positively correlated with the temperature in the range of 0 **°**C–50 **°**C and corresponded to the Arrhenius relation^19^. In addition, either raising the temperature or increasing the oxidant concentrations has been found to facilitate the catalytic etching process by reducing the reaction barrier of hole injections through metal/Si interfaces, which speeded up the formation rate of Si nanowires^18^. However, previous studies showed that the slight increase of temperature upon etching process up to 60 **°**C caused the significant generation of nanopores within Si nanowires, dramatically changing their luminescent properties^20^. The addition of H_2_O_2_ oxidants over 0.3 M led to the formation of nanowires with rough sidewalls, and even altered the nanowire geometries because the involved Ag catalysts were unable to sustain their original shapes in such concentrated H_2_O_2_ solutions^21, 22^. In addition, using different solvents, such as ethylene glycol, also resulted in the growth of porous nanostructures upon MaCE process^23^.

These inevitable involvements of nanopores on nanowire formation, normally appearing on the nanowire sidewalls with random distributions^19–24^, significantly altered the electric, optoelectric and mechanic properties of as-formed nanowire arrays. In the aspect of reducing process time, it would be highly desirable to find a reliable route to stably increase the etching rate of MaCE without the introduction of unwanted pores created on nanowire surfaces. Therefore, in this study, a novel incorporation of conductive substrates attached on the Si substrates with a conventional MaCE process was explored to overcome such remaining challenges, which could effectively enhanced the formation rates of Si nanowire arrays in a facile manner. Investigations on etching kinetics, surface morphologies and wetting characteristics were performed to understand the role of back substrates on MaCE reaction.

## Methods

### Synthesis of aligned Si nanowire arrays

(100)-oriented, single-crystalline and lightly-doped Si substrates (resistivity = 1–10 Ω cm) with either p-type or n-type configurations were utilized as the starting samples. Prior to etching experiments, Si substrates with fixed size of 2 cm by 2 cm were cleaned by the regular ultrasonication in acetone, isopropyl alcohol (IPA) and deionized water (DI) water for several times. After drying with gentle N_2_ gas, nonconductive adhesive tape (Kapon) or the various layers of conductive carbon type (Ted Pella, Inc.), were carefully attached onto the back side of Si substrates. In addition, sheet resistances of back substrates were evaluated with conventional four-point resistivity system. Etching of Si was conducted by two-steps MaCE process. In the first step, the as-prepared Si substrates were dipped in the mixed solution containing 0.005 M of AgNO_3_ and 4.6 M of HF at 25 **°**C for 10 s. After that, Ag-deposited Si substrates were rinsed with DI water and then dried by N_2_ gas. In the second step, Si substrates with Ag loadings were immersed in the etching solutions containing 0.3 M of H_2_O_2_ and 4.6 M of HF at 25 **°**C for 10 min. After etching process, the nanowire-based samples were rinsed with DI water and dried in the stream of N_2_ gas. The residual Ag nanoparticles were removed by dipping in the concentrated HNO_3_ (65%) for 5 min and rinsed several times.

### Characterizations

Surface Morphologies of Si nanowires were investigated with scanning electron microscope (SEM, Hitachi S-4800). Wetting properties of samples were characterized with contact angle measurements (Theta Lite) from imaging a drop of water with 1 μl in volume. Photoluminescent (PL) measurements were conducted at room temperature, using a light source of light-emitting diode lamp with the output power of 780 mW and center wavelength of 365 nm.

## Results and Discussion

**Figure 1:**
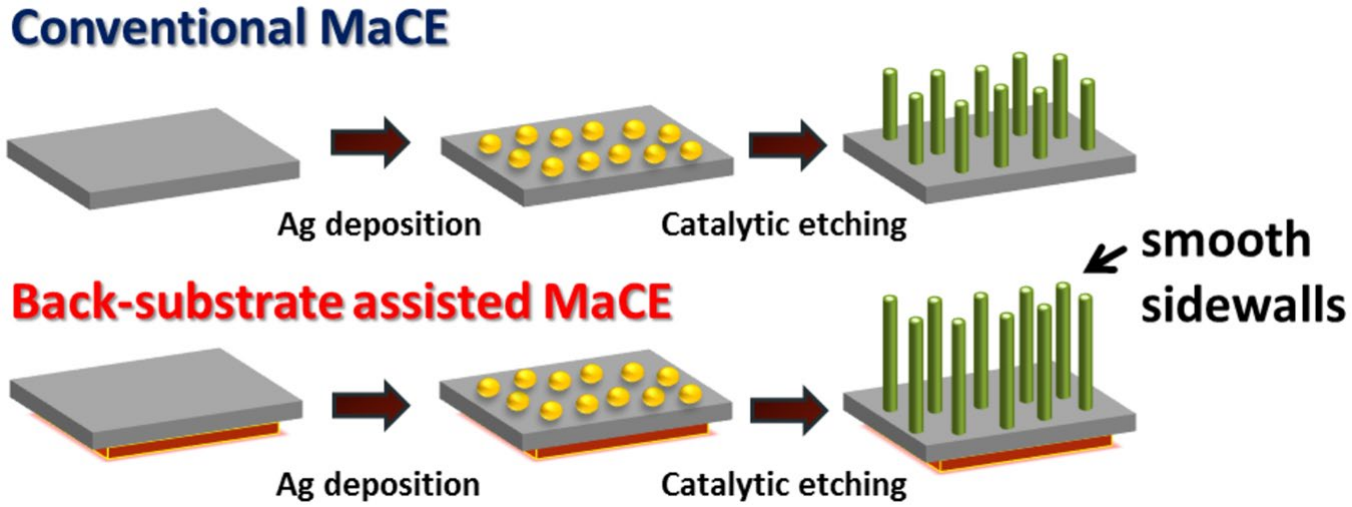
Schematic illustrations for the fabrication procedures of both conventional MaCE and back-substrate assisted MaCE. Notice that the formed nanowires using the modified MaCE method possessed the smooth sidewalls.

Figure 1 presented the distinct processing strategies of MaCE technique. With the utilization of conventional MaCE process, etching experiment was conducted on the Ag-loaded Si (100) substrate in the presence of mixing solutions containing two major reactants, including H_2_O_2_ as oxidants and HF as etchants. Due to the nature of Si crystallographic structures responding to different back-bond strengths, dissolution of Si was preferentially occurred along 〈100〉 directions while reactants in solutions were in contact with Ag/Si interfaces^25, 26^. Through the continuous sink of metal nanoparticles into Si substrates as etching reaction proceeded, 〈100〉-oriented Si nanowires were therefore created. In our experiment, conductive and nonconductive films were initially adhered on the back side of Si substrates prior to etching process. Effects of such incorporation of back substrates with MaCE processing were carefully investigated on both morphological and structural aspects. Notice that the formed nanowires obtained from back-substrate assisted MaCE method possessed the unexpectedly smooth sidewalls, which will be discussed later.

**Figure 2:**
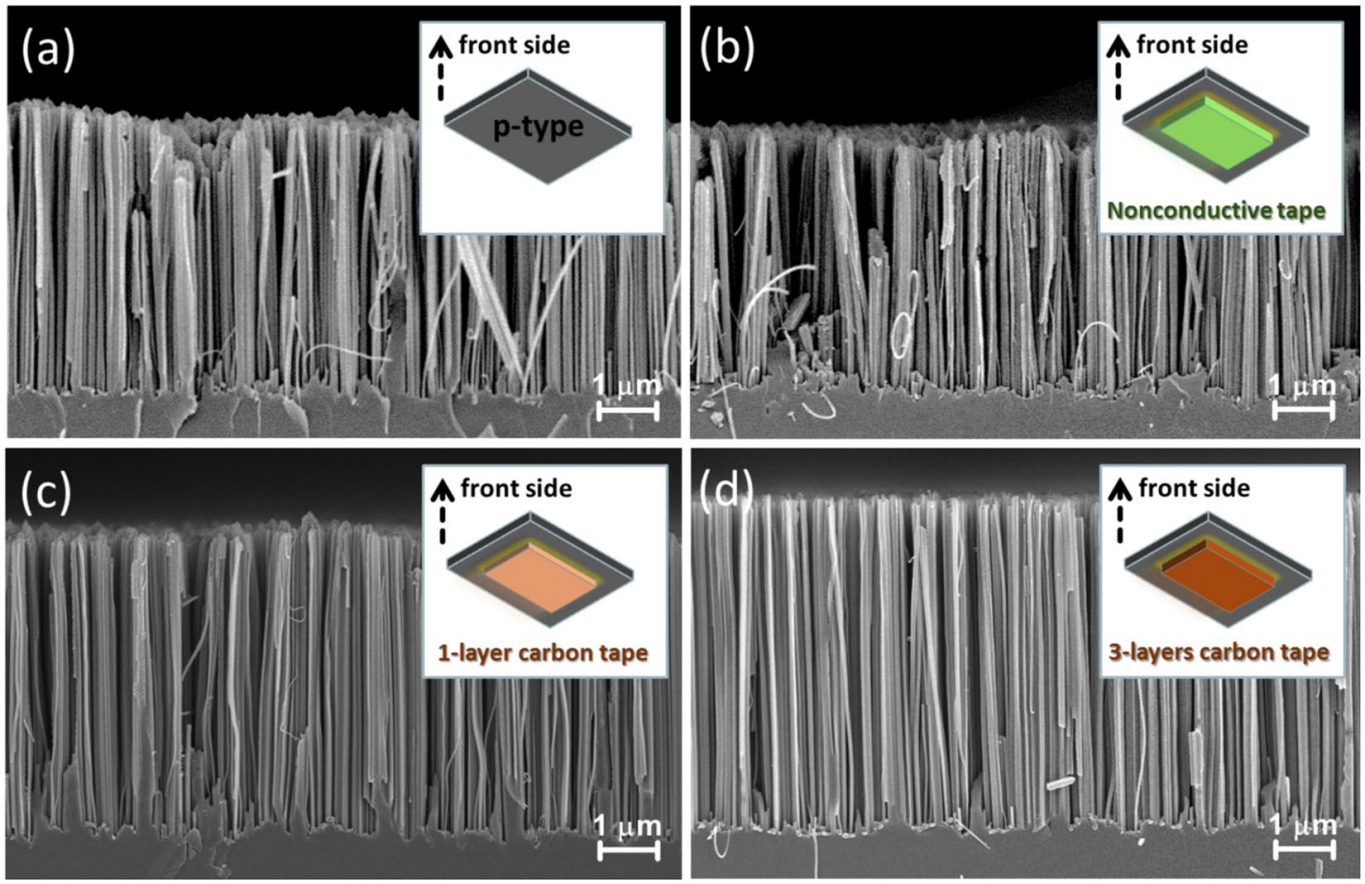
Cross-sectional SEM images of aligned Si nanowire arrays obtained from (**a**) conventional MaCE and back-substrate assisted MaCE with back substrates of (**b**) nonconductive tape, (**c**) one-layer and (**d**) three-layer conductive carbon tapes. The corresponding sample configurations are depicted in the insert figures, respectively.

Figure 2 summarized the SEM observations obtained from applying various back substrates on the result of MaCE reaction, including the case of nonconductive additive tape [Fig. 2(b)], one-layer conductive carbon tape [Fig. 2(c)] and three-layer conductive carbon tape [Fig. 2(d)], respectively. The corresponding configurations of samples were also depicted in the insert figures, respectively. Apart from that, Fig. 2(a) demonstrated the etching results upon conventional MaCE process. By comparing the SEM results shown in Fig. 2(a,b), it could be clearly found that the lengths of formed nanowire arrays from bare Si substrates and Si substrates attached with nonconductive additive tape were almost identical. Interestingly, with the attachment of conductive tape on the back side of Si substrates, the monolithic increase of nanowire lengths from one-layer to three-layer carbon tape could be apparently observed. These features strongly implicated that the presence of conductive back substrates, under the processing of MaCE reaction, acted an explicit role on the dynamic modulation of etching phenomena.

**Figure 3:**
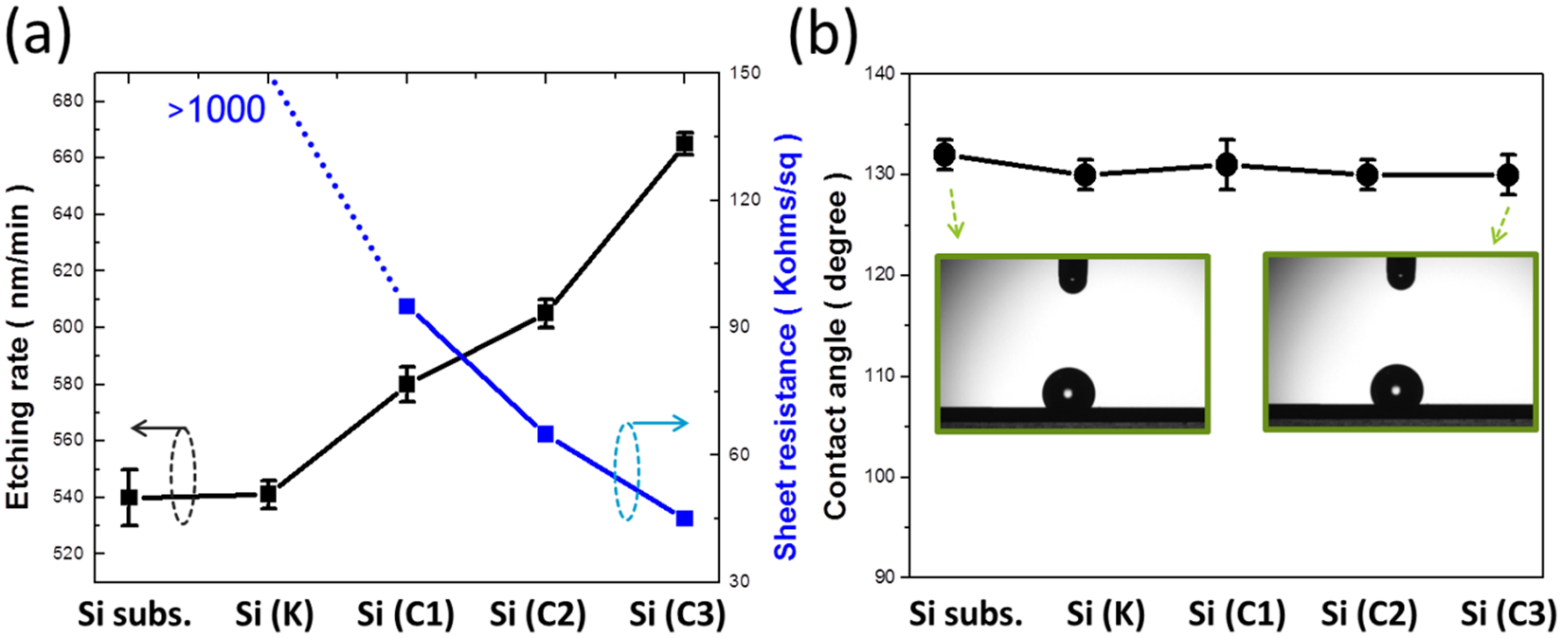
(**a**) Dependence of etching rates on the types of Si substrates: bare Si substrate (Si subs.), Si substrate with nonconductive tape (Si (K)), Si substrate with one-layer (Si (C1)), two-layer (Si (C2)) and three-layer (Si (C3)) conductive carbon tapes. The corresponding sheet resistances of back substrates were also presented. (**b**) Contact-angle measured results of Si nanowire arrays formed by various types of Si substrates.

To further explore the underlying changes of etching rates, the dependence of etching rates on the types of back substrate in relation to the corresponding sheet resistance were graphed in Fig. 3(a). The dramatic increase of etching rates upon MaCE process could be found when applying the conductive back substrates, with the measured values of 540 ± 9 nm/min from bare Si substrate, 542 ± 5 nm/min from Si substrate with nonconductive tape, 580 ± 6 nm/min from Si substrate with one-layer carbon tape, 605 ± 5 nm/min from Si substrate with two-layer carbon tape and 665 ± 4 nm/min from Si substrate with three-layer carbon tape. For each experimental condition, the similar experiments were conducted for 10 times and the average etching rates along with the ranges of deviation were measured and recorded in Fig. 3(a), respectively. It was fond that the deviations of etching rates were less than 5% for all cases with the employment of back substrates regardless of their sheet resistances. Furthermore, the resulting formation rates of nanowire arrays were found to inversely correlate with the sheet resistances of applied back substrates. These results could be evidently supported by comparing the etching rates obtained from three various layers of carbon types, as presented in Fig. 3(a). In addition to the etching kinetics, surface topographies of synthesized nanowires should be further examined. The wetting features of nanostructures could be characterized by measuring the contact angle of nanowire samples, and the average values along with the ranges of deviation were presented in Fig. 3(b). The comparable values of contact angles within the tiny range of 130°–132° could be found from the nanowires with or without the employment of back substrates, evidencing that no obvious topographic changes from these nanowire arrays. For applying MaCE techniques on practical needs, high-speed formation rate of uniform Si nanowire arrays was particularly decisive as it closely related to the fabrication throughput, and even allowed the effective reduction of manufacturing cost. In this regard, back-substrate assisted MaCE technique may meet such practical requirement for the advanced manufacture of Si nanowire-based devices and applications.

**Figure 4:**
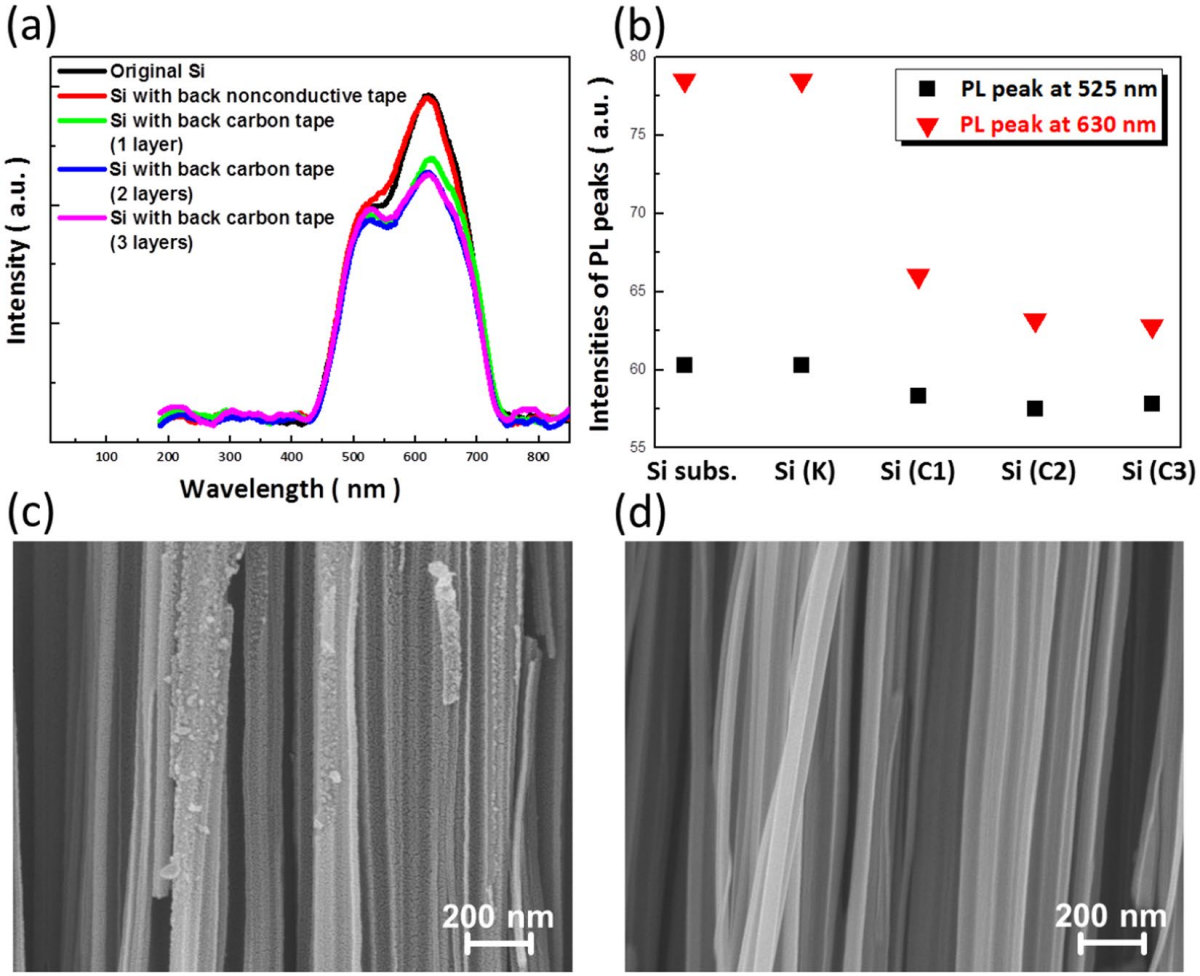
(**a**) PL spectra and (**b**) intensities of two pronounced PL peaks from Si nanowire arrays prepared with conducting MaCE process on various types of Si substrates. High-magnification SEM images of Si nanowires fabricated by (**c**) conventional MaCE and (**d**) back-substrate assisted MaCE with three layers of conductive carbon tape as back substrates.

It has been reported that the light-emitting characteristics of Si nanowires were strongly associated with their surface structures. Specifically, the red-light emissions were contributed from the involvement of porous features, and the wideband blue/green-light emissions were originated from the rough surfaces of nanowire sidewalls^27^. From the PL measurements of nanowire structures obtained from conventional and back-substrate assisted MaCE process, the essence of morphological structures correlated with their PL emissions could be identified, as demonstrated in Fig. 4(a). It was evident that there were two pronounced PL peaks appearing at all nanowire samples, with a weak peak (525 nm in wavelength) and a predominant peak (630 nm in wavelength) contributed from rough and porous configurations of formed nanowires, respectively. Notably, the PL intensities at 680 nm were strongly diversified responding to the introduction of back substrates upon nanowire formation. These characteristics could be further unveiled in Fig. 4(b), where the long-wavelength PL peaks centered at 525 nm remained unchanged regardless of the employment of back substrates; the explicit reduction of peak intensities at 630 nm was found when applying various layers of conductive carbon tapes as back substrates. These findings suggested that the presence of back substrates under MaCE reaction acted an important role on preventing the creation of nanopores from the excess hole injections into Si in random orientations. High-magnification SEM images shown in Fig. 4(c,d) again confirmed that the reduced pore formation was evolved on nanowire surfaces during the back-substrate assisted MaCE process. In fact, porous features of Si nanowires might cause strong charge recombination of photogenerated carriers in photovoltaic cells, thus degrading the resulting conversion efficiency of light-to-electricity. In this aspect, back-substrate involved MaCE could potentially offer a desired pathway for generating nanowires on photovoltaic applications.

**Figure 5:**
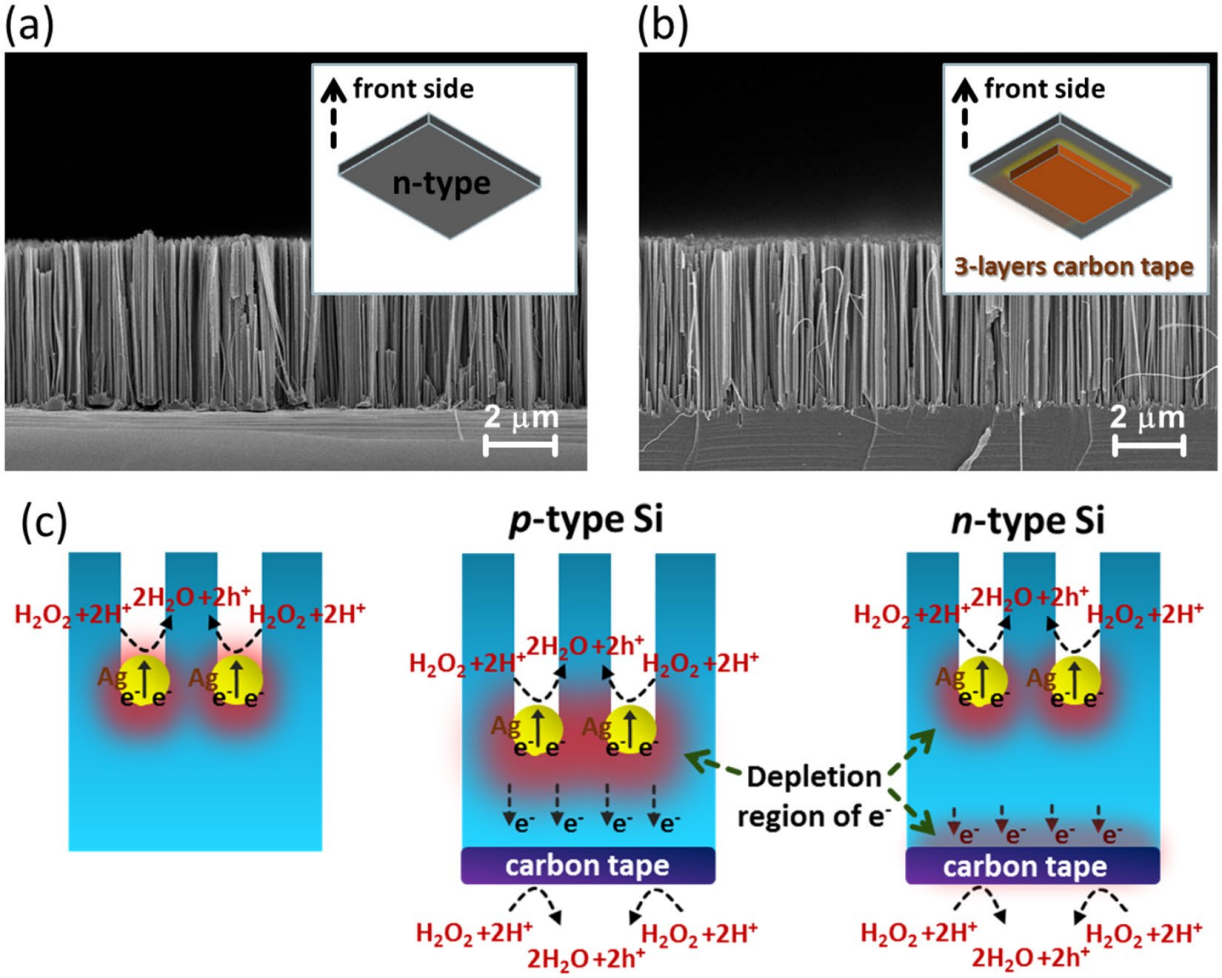
Cross-sectional SEM images of aligned Si nanowire arrays obtained from catalytic etching of n-type Si: (**a**) conventional MaCE and (**b**) back-substrate assisted MaCE with three-layer conductive carbon tape as back substrates. (**c**) Schematic illustrations for the distinct formation mechanisms of MaCE process: Si substrates (p-type or n-type) without conductive back substrates, Si substrates (p-type) and Si substrates (n-type) with conductive back substrates.

To understand the role of back substrates on nanowire formation, the additional experiments were conducted by etching the Si substrates with n-type configuration. Notice that the range of doping concentrations of n-type Si used in this test were exactly similar with that of p-type Si and all of the experimental conditions were carefully maintained. The representative results from the comparisons of samples made by MaCE process without back substrates [Fig. 5(a)] and with three-layer conductive back substrates [Fig. 5(b)] showed the identical lengths of fabricated Si nanowire arrays. Supported by these findings, together with detailed etching kinetics and structural investigations, the possible reaction mechanism of back substrates involved in MaCE process could be speculated, as illustrated in Fig. 5(c). Without the attachment of back substrates on Si, dissolution of Si atoms was simply sustained by a single pathway. H_2_O_2_ oxidants were preferentially decomposed at Ag surfaces through the attraction of free electrons from Ag nanoparticles.

Meanwhile, electrons of Si were extracted out right through Ag nanoparticles due to the fact that the redox potential of H_2_O_2_ was lower than the energy level of valence band in Si with the presence of HF acids^18, 28, 29^. It implicated the oxidation of Si at Ag/Si interfaces and the etching process was accomplished by dissolving Si oxide in HF-contained solutions. This dynamic process stayed valid when introducing the nonconductive back substrates since the entire electrochemical reaction did not altered. Nevertheless, incorporation of conductive back substrates with similar H_2_O_2_/HF condition at the back side of p-type Si introduced an additional pathway for the extraction of electrons from Si. Namely, in addition to the direct extraction of electrons at Ag/Si interfaces, the decomposition of H_2_O_2_ oxidants could also take place at the surfaces of carbon tape exposed to the solutions. This created the concentration gradient of electrons in the vicinity of carbon tape, thus driving the diffusion of electrons toward the back side of p-type Si. The involved two pathways for electron extraction favored the depletion of electrons close to the etching front, which speeded up the localized oxidation of Si at Si/Ag interfaces, eventually increasing the etching rate of MaCE process, as evidenced in Figs 2 and 3(a). It has been reported that the excessive holes generated by H_2_O_2_ oxidants may lead to the porosification process that prevailed on the sidewalls or the top surfaces of nanowires^21–23, 30^. With the assistance of back substrates, such porosification features could be slowed down or even diminished because the additional routes driven by back substrates assisted to rectify the diffusions of excessive charges that reduced the possibility for pore formation upon MaCE process.

It should be noted that the primary oxidation of Si was initiated by the catalytic Ag nanoparticles rather than the conductive back substrates, so the similar nanowire arrays could be always found as the final etching morphology was still determined by the defined Ag nanoparticles. Such accelerated etching mechanism could be further supported when applying the conductive back substrates on n-type Si. The depletion regions of electrons introduced by the effect of back substrates were either limited closely at Ag/Si or carbon-tape/Si interfaces due to substantial concentration of electrons existed in n-type Si. Therefore, etching of Si was still kinetically dominated by the Ag-assisted catalytic dissolution of Si and reflected the unchanged etching rates in comparison with the result of conventional MaCE process, as evidenced in Fig. 5(a,b).

## Conclusions

In conclusion, a novel MaCE technique with the assistance of back substrates has been developed, which visualized the large-area uniformity, well-structure regularity and high-speed etching for the generation of vertically aligned Si nanowire arrays. By examining etching kinetics, morphologies, wetting behaviors and PL signals, the involving mechanism associated with the role of back substrates on MaCE process were clarified. These advanced etching techniques, along with improved formation rate of Si nanowires, were anticipated to be potential for many applications including energy conversion, thermoelectrics, sensing and other functional devices.
